# Dietary Intake and Plasma Levels of Choline and Betaine in Children with Autism Spectrum Disorders

**DOI:** 10.1155/2013/578429

**Published:** 2013-12-16

**Authors:** Joanna C. Hamlin, Margaret Pauly, Stepan Melnyk, Oleksandra Pavliv, William Starrett, Tina A. Crook, S. Jill James

**Affiliations:** ^1^Department of Dietetics and Nutrition, University of Arkansas for Medical Sciences, Little Rock, AR 72202, USA; ^2^Department of Pediatrics, University of Arkansas for Medical Sciences, Little Rock, AR 72202, USA; ^3^Department of Pediatrics, University of Arkansas for Medical Sciences, Arkansas Children's Hospital Research Institute, 13 Children's Way Slot 512-41B, Little Rock, AR 72202, USA

## Abstract

Abnormalities in folate-dependent one-carbon metabolism have been reported in many children with autism. Because inadequate choline and betaine can negatively affect folate metabolism and in turn downstream methylation and antioxidant capacity, we sought to determine whether dietary intake of choline and betaine in children with autism was adequate to meet nutritional needs based on national recommendations. Three-day food records were analyzed for 288 children with autism (ASDs) who participated in the national Autism Intervention Research Network for Physical Health (AIR-P) Study on Diet and Nutrition in children with autism. Plasma concentrations of choline and betaine were measured in a subgroup of 35 children with ASDs and 32 age-matched control children. The results indicated that 60–93% of children with ASDs were consuming less than the recommended Adequate Intake (AI) for choline. Strong positive correlations were found between dietary intake and plasma concentrations of choline and betaine in autistic children as well as lower plasma concentrations compared to the control group. We conclude that choline and betaine intake is inadequate in a significant subgroup of children with ASDs and is reflected in lower plasma levels. Inadequate intake of choline and betaine may contribute to the metabolic abnormalities observed in many children with autism and warrants attention in nutritional counseling.

## 1. Introduction

Autism is a complex, behaviorally-defined neurodevelopmental disorder characterized by significant impairments in social interaction, verbal and nonverbal communication, and by restrictive, repetitive, and stereotypic patterns of behavior. The Centers for Disease Control estimates that the current prevalence of autism spectrum disorders (ASD) in the United States is 1 in 110 children [1]. Nutritional screening and assessment of children with ASDs is an important clinical consideration for several reasons. First, these children often exhibit nutrition-related medical issues including gastrointestinal discomfort, bowel inflammation, diarrhea, constipation, and acid reflux [1]. Abnormal sensory processing can affect taste and texture perception leading to food avoidance and restricted food intake in many children with ASD. “Insistence on sameness” and compulsive repetitive behaviors reinforce rigid dietary preferences and lead to a limited food repertoire [2]. Finally, accumulating research indicates that nutrient metabolism and requirements may be altered in some children with ASDs compared to typically developing children [3–5]. Thus, children with ASDs have multiple risk factors that may increase the prevalence of nutrient deficiencies in this population.

Metabolic abnormalities reported in children with ASDs have primarily involved folate-dependent one-carbon metabolism. Paşca et al. reported hyperhomocysteinemia and abnormal methionine metabolite levels in children with AD and PDD-NOS [6]. They also noted an increased prevalence of the C677T MTHFR polymorphism in children with AD. Polymorphisms in this pathway limit folate availability and increase the need for other interdependent metabolites including choline and betaine [7]. In addition, James et al. found that children with ASDs had significantly lower plasma concentrations of methionine, S-adenosylmethionine (SAM), cystathionine, cysteine, and total glutathione (GSH) and significantly higher concentrations of S-adenosylhomocysteine (SAH), adenosine, and oxidized glutathione (GSSG) when compared to age-matched control children [8–10]. These metabolic abnormalities can lead to compromised methylation (SAM/SAH) and antioxidant/detoxification capacity (GSH/GSSG). In one study, low plasma SAM/SAH was associated with DNA hypomethylation and low plasma GSH/GSSG was associated with biomarkers of protein oxidative damage (3-nitrotyrosine, 3-chlorotyrosine) and DNA oxidative damage (8-oxodeoxyguanine) [10]. Rose et al. found a similar decrease in GSH/GSSG and oxidative damage in postmortem brain samples from individuals with autism suggesting that oxidative stress and damage may be a systemic issue in some children with autism [11].

**Figure 1:**
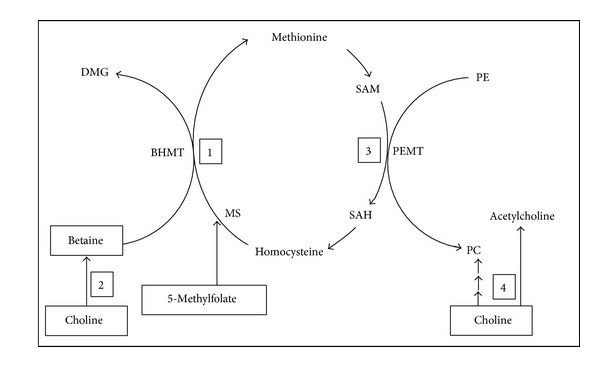
Interrelated and interdependent pathways of (1) folate- and betaine-dependent methionine resynthesis from homocysteine utilizing folate-dependent methionine synthase (MS) and betaine-dependent betaine : homocysteine methyltransferase (BHMT); (2) choline-dependent betaine synthesis; (3) phosphtidylethanoloamine methyltransferasse (PEMT) conversion of phosphatidylethanolamine (PE) to phosphatidylcholine (PC); and (4) choline-dependent synthesis of PC and acetylcholine.

Choline, betaine, and folate are interchangeable sources of one-carbon units. As shown in Figure 1, the metabolism of choline intersects with folate-dependent one-carbon metabolism as an alternate pathway for methionine synthesis, especially when folate availability is limited. Choline is the precursor for betaine and methyl groups derived from betaine which are used for SAM-dependent methylation reactions including the synthesis of membrane phosphatidylcholine (PC). In this way, choline indirectly serves as a precursor for the synthesis of membrane phospholipids that are essential for normal membrane fluidity, signal transduction, membrane transport and integrity [12, 13]. Choline is also a precursor for the synthesis of acetylcholine (ACh), an important neurotransmitter in both the central and autonomic nervous systems. In the central nervous system, ACh is an important neuromodulator of sensory perceptions and inducer of REM sleep and is important for sustaining attention [14]. Finally, as a methyl donor for SAM synthesis, choline deficiency has been shown in animal models to contribute to global DNA hypomethylation and epigenetic abnormalities [15]. Low plasma SAM levels and DNA hypomethylation have also been shown to be present in children with autism [10].

Choline was recognized by the Institute of Medicine (IOM) as an essential nutrient in 1998 [16]. Good dietary sources of choline include eggs, liver, beef, chicken, fish, milk, cruciferous vegetables, beans, and peanuts, whereas betaine is primarily obtained from wheat bran, wheat germ, and spinach [9, 10]. Notably, betaine intake has been negatively associated with the Western diet high in meat, sugar, and fat [11]. Zeisel [17] observed the following symptoms when healthy individuals consumed a choline deficient diet: (1) hepatic steatosis, (2) muscle damage, (3) DNA damage, and (4) changes in lymphocyte gene expression. In addition, low plasma choline levels have been associated with increased anxiety [18].

Although choline and its metabolites are important contributors to normal folate-dependent one-carbon metabolism, dietary intake and plasma levels of these nutrients have not been investigated in the ASD population. Therefore, the purpose of the study was to determine whether age-specific dietary intake of these nutrients was within the adequate range by national standards and whether dietary intake was correlated with plasma levels in a subset of these children.

## 2. Subjects and Methods

### 2.1. Study Participants

Nutritional data on choline and betaine intake from food was obtained from 288 children with ASDs who participated in the Autism Intervention Research Network for Physical Health (AIR-P) Study on Diet and Nutrition in Children with Autism and they were recruited from four national sites including Pittsburg, Pennsylvania, Little Rock, Arkansas, Rochester, New York, and Denver, Colorado. A subgroup of 35 of the 288 ASD participants and 32 control participants whose parents consented to a blood draw participated in an ancillary study in which plasma choline metabolites were measured and compared between groups. Inclusion criteria for the ASD group included children 2–11 years of age with clinical diagnoses of an ASD based on the *Diagnostic and Statistical Manual IV* criteria and the Autism Diagnostic Observation Schedule (ADOS). Control participants were 3–10 years of age and had no medical history of behavioral or neurological abnormalities, as determined by parent report, and were control participants in an ongoing NICHD-sponsored study of children with autism (SJJ: R011HD051873). Control children were age and sex-matched to the case children for the plasma analysis and were limited to parents who agreed to have their child's blood drawn. The study protocols and informed consents were approved by the Institutional Review Boards at each site where data were collected.

### 2.2. Dietary Data

Three-day food records were collected from caregivers of the participants in the ASD group (*n* = 288). Trained personnel used a standardized method to instruct caregivers on recording all foods, beverages, and supplements consumed by the participants for three consecutive days, including one weekend day. Completed records were returned to each site for review and caregivers were contacted if information was missing or unclear. Records from each site were sent to Rochester, New York for analysis using the Nutrition Data System for Research (NDSR) software versions 2009 and 2010, developed by the Nutrition Coordinating Center (University of Minnesota, Minneapolis, MN). Individual food intake results were based on the mean intake from all three days of data collection.

### 2.3. Plasma Data

Plasma concentrations of choline and betaine were obtained from 67 participants (35 with ASD and 32 controls) whose parents consented to the blood draw. Participants were instructed to fast 12 hours prior to the blood draw. The maximum blood drawn was 25 mL per participant. The blood sample was obtained within two weeks of the completion of the 3-day food record. After samples were obtained and deidentified, they were sent to the Autism Treatment Network/Intellectual & Developmental Disabilities Research Center (ATN/IDDRC) Biorespository in Denver, Colorado for storage. A 250 uL aliquot was sent to the Autism Genomics Laboratory in Little Rock, Arkansas for analysis. Choline and betaine concentrations were measured using a Dionex High Performance Liquid Chromatography-Ultraviolet System coupled to an electrospray ionization (ESI) tandem mass spectrometer using Thermo-Finnagen LCQ. Samples of 30 *μ*L were deprotenized with three volumes of acetonitrile and further analyzed using normal phase chromatography on silica gel column. It was equilibrated with a mixture of 15 mmol/L ammonium formate and acetonitrile in a ratio of 25 : 75 by volume. It was eluted with a linear gradient of increasing proportions of ammonium formate, as described in greater detail in Holm et al. [19].

### 2.4. Statistical Analysis

Statistical analyses were conducted using SPSS (version 21.0) and Excel software (Microsoft Office 2007; Microsoft Corp., Redmond, WA). Descriptive statistics were used to describe the study participants' demographic characteristics. Means, standard deviations, and ranges were used to describe the dietary intake of the ASD group. Pearson's product-moment correlation coefficients were used to test the relationships between dietary intake and plasma levels of choline and betaine in the ASD group. Student's *t*-tests were used to determine if differences existed in plasma concentrations between groups. Statistical significance was set at 0.0.

## 3. Results

### 3.1. Participant Characteristics

Among the 288 ASD participants, 86.1% were male, 25.7% (74) were in the 1–3-year-age category, 61.5% (177) were in the 4–8-year-age category, and 12.8% (37) were in the 9–11-year-age category. Greater than 90% of the participants were Caucasian. Within the subgroup of children evaluated for plasma and dietary intake of choline and betaine, 11 of the 35 children (32%) were 1–3 years old, 19 children (54%) were 4–8 years old, and 5 children (14%) were 8–11 years of age. Anthropometric data from the ASD subgroup (*n* = 35) and control group (*n* = 32) indicated that 27% of children in the ASD group were in the overweight and obese categories compared to 23% in the control group. Additionally, fewer children in the ASD group were classified as underweight compared to the control group (6% versus 10%, resp.).

### 3.2. Dietary Intake of Participants with ASD

Dietary intake data is based on three-day food records of the 288 ASD participants analyzed at the time of paper preparation. As shown in Table 1, choline intake was below the AI for more than 69% in all age categories. The proportion of children with intake below the AI increased progressively with age (range 69–93%). No dietary reference intake levels have been established for betaine; however, the average US adult betaine intake has been estimated to be ~5 mg/kg/day [20, 21]. The mean betaine intake in the children with autism was ~4.6 mg/kg/day across all age groups. However, the percent of children whose intake was less than 3.5 mg/Kg/day was 30% in the 1–3 yr age group, 23% in the 4–8 yr age group, and 18% in the 9–11 yr age group.

### 3.3. Relationships between Dietary Intake and Plasma Concentrations of Choline and Betaine in ASD Group

**Figure 2:**
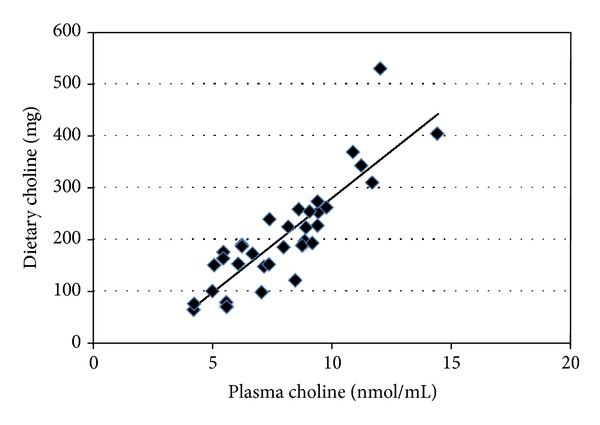
Correlation between dietary intake and plasma choline concentrations in children with ASD (*n* = 35). *r* = 0.86 and *P* ≤ 0.001 using Pearson's product-moment correlation coefficient. ASD: autism spectrum disorder.

**Figure 3:**
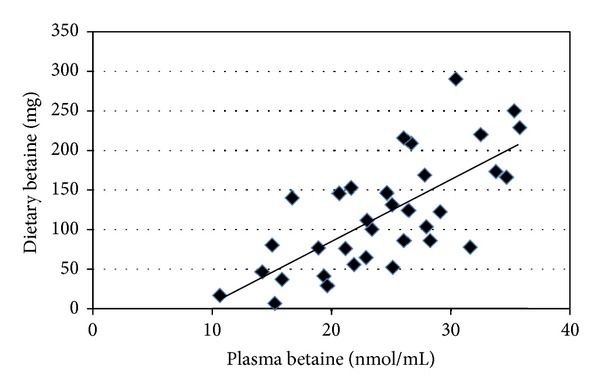
Correlation between dietary intake and plasma betaine concentrations in children with ASD (*n* = 35). *r* = 0.67 and *P* ≤ 0.001 using Pearson's product-moment correlation coefficient. ASD: autism spectrum disorder.

Relationships between dietary intake and plasma concentrations of choline and betaine in the ASD cohort (*n* = 35) were investigated using Pearson's product-moment correlation coefficients. There was a strong, positive correlation between dietary intake and plasma choline concentrations: *r* = 0.86, *n* = 35, and *P* < 0.001, with low intake associated with low plasma choline concentrations (Figure 2). Similarly, dietary intake and plasma betaine concentrations showed a strong, positive correlation: *r* = 0.67, *n* = 35, and *P* < 0.001, with low dietary intake associated with low plasma betaine concentrations (Figure 3).

### 3.4. Comparison of Plasma Metabolite Concentrations in ASD and Control Groups

**Figure 4:**
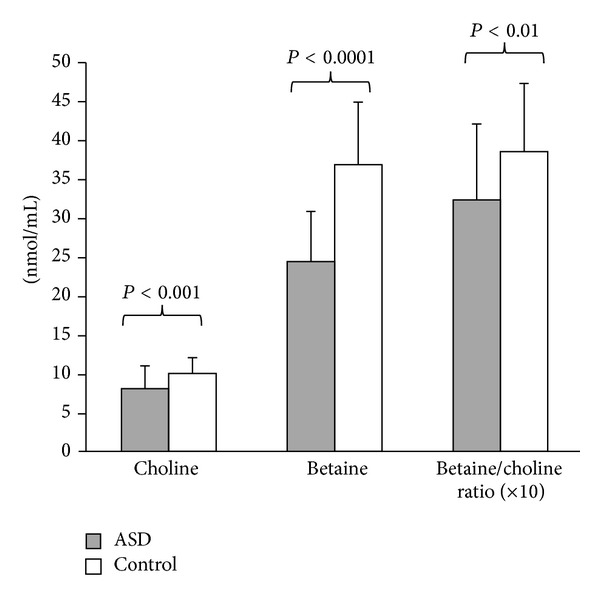
Plasma levels of choline, betaine, and the betaine/choline ratio in children with autism compared to age-matched controls.

A comparison of plasma concentrations of choline and betaine was made between the ASD cohort (*n* = 35) and the control group (*n* = 32) and is presented in Figure 4. Student's *t*-test demonstrated that participants in the ASD group had significantly lower plasma concentrations of choline and betaine compared to the control group (*P* < 0.001) as well as a significant decrease in the betaine : choline ratio.

## 4. Discussion

The results of the AIR-P study of diet and nutrition in children with autism demonstrate for the first time that the majority of children with ASDs between 3 and 11 years of age consume inadequate amounts of dietary choline. A strong correlation between choline and betaine dietary intake and plasma levels was observed in a subset of these children suggesting that the choline-betaine-homocysteine pathway for methionine synthesis may be compromised. The significant decrease in choline : betaine intake ratio presented in Figure 4 is consistent with this possibility. Research studies have shown that insufficient dietary folate increases requirement for choline and betaine-derived methyl groups and conversely, choline and betaine deficiency increases the requirement for folate-derived methyl groups [17]. Thus, dietary deficits in both pathways for methionine synthesis may be compromised in children with ASDs and additively contribute to the low methionine and SAM levels previously reported in these children [8–10]. Importantly, reduced synthesis of SAM, the major intracellular methyl donor, can lead to DNA hypomethylation and epigenetic abnormalities associated with abnormal gene expression, genomic imprinting, and genomic instability [22]. Significant decreases in plasma methioinine and SAM associated with DNA hypomethylation have been reported in children with ASDs relative to age-matched control children [10].

It is not known whether supplemental choline or betaine would increase methionine and SAM synthesis in children with autism. However, works by Atkinson et al. [23] and Innis et al. [24] support the positive effects of choline and betaine in other studies. Atkinson et al. conducted a randomized crossover study in healthy males (*n* = 8) that measured betaine and homocysteine concentrations after consuming meals or supplements containing choline or betaine. They found that betaine from meals and supplements acutely increased plasma betaine. Additionally, both betaine and choline helped alleviate the rise in homocysteine concentrations following a postmethionine load. Innis et al. found that a choline supplement in children with cystic fibrosis resulted in significant increased methionine, SAM, the SAM/SAH methylation ratio, and the GSH/GSSG redox ratio. Because the metabolic profile of children with ASDs is similar to that observed in children with cystic fibrosis, it is possible that choline supplementation may similarly improve methylation status in children with ASDs.

Consistent with low choline status, El-Ansary et al. [25] found that phosphatidylethanolamine, phosphatidylserine, and phosphatidylcholine were significantly lower in a group of Saudi Arabian children with ASDs (*n* = 25) compared to a control group (*n* = 16). They suggested that the lower levels of these phospholipids could be related to oxidative stress and inflammation. Similarly, James et al. found decreased plasma levels of cysteine, glutathione, and the ratio of reduced to oxidized glutathione (GSH/GSSG) in children with ASDs compared to a control group, indicating that some children with ASDs have reduced antioxidant capacity and evidence of oxidative stress [8]. Other researchers have reported higher homocysteine levels in children with ASDs [6] which is important to consider since choline and betaine have been shown to reduce these levels, especially when given in addition to methionine. In addition to inadequate intake of choline and betaine, the AIR-P study of diet and nutrition in children with autism reported that calcium, vitamin E, vitamin D, and fiber intake are also inadequate when compared to NHANES normative data [2].

A final consideration is the role of choline deficiency in brain development, memory, and anxiety. In rodent models, multiple studies have shown that choline deficiency and supplementation affect neurodevelopment. The offspring of choline-supplemented pregnant rodents have improved visuospatial and auditory memory and perform better in behavioral tests, whereas choline deficiency seems to have the opposite effect [26, 27]. Fewer studies have been done in humans, although the elderly and patients with Alzheimer's disease have reduced levels of free choline and phosphatidylcholine in the brain [28, 29]. A recent large population based study of 5,918 men and women participating in the Hordaland [18] Health Study, found that low plasma choline concentrations were significantly associated with higher anxiety levels. Behavioral alterations associated with low plasma choline levels in children with ASDs warrant further research consideration.

The present study had several possible limitations. First, it is possible that parents who consented to participate may have been more concerned about nutrition and feeding behaviors in their children such that their dietary patterns might be different from the general population of children with ASD. We were unable to make comparisons regarding the diets of the unaffected control children since food records were only collected for children with ASDs. Also, it is unclear if the differences observed in plasma concentrations between case and control groups are reflective of their dietary intake or abnormal metabolism or both. While the adequacy of choline intake was determined using the standard AI levels, a component of the dietary reference intakes that is intended for healthy individuals, it is uncertain if these standards can be applied to children with ASDs, especially since abnormalities in nutrient metabolism have been found in these children.

## 5. Conclusions

In summary, choline plays an essential role as a methyl-group donor in the synthesis of the membrane phospholipid components of cell membranes as well as in the synthesis of the neurotransmitter acetylcholine. The data in the AIR-P diet and nutrition study indicate that 69 to 93% of children with ASDs consumed diets that were inadequate in choline. Importantly, low choline and betaine intake were associated with low plasma levels of these nutrients suggesting that there could be functional consequences related to folate and phospholipid metabolism. Future research should consider whether these metabolic imbalances can be corrected with dietary counseling or supplement interventions and whether metabolic improvement is associated with improvement in some behavioral symptoms.

## Figures and Tables

**Table 1:** Mean dietary intake of choline and betaine in children with ASD (*n* = 288).

Age	Choline intake (mg) (mean ± SE)	AI^a^ for choline (mg)	Choline intake less than AI^a^ (% children)	Betaine intake^b^ (mg/kg) (mean ± SE)	Betaine intake less than 3.5 mg/kg (% children)
1–3 y (*n* = 72)	176 ± 10	200	68.7%	4.6 ± 0.18	30%
4–8 y (*n* = 178)	182 ± 5	250	84%	4.7 ± 0.47	23%
9–11 y (*n* = 38)	238 ± 14	375	93.2%	4.6 ± 0.20	18%

Note: ^a^AI: adequate intake; ^b^average adult betaine intake = ~5 mg/kg [14, 15].

## Abbreviations

ASDs: Autism spectrum disorders
AIR-P: Autism Intervention Research Network for Physical Health
NICHD: National Institute of Child Health and Human Development
DMG: Dimethylglycine
AI: Adequate intake
AD: Autistic disorder
PDD-NOS: Pervasive developmental disorder not-otherwise specified
MTHFR: Methylenetetrahydrofolate reductase
SAM: S-adenosylmethionine
GSH: Total glutathione
SAH: S-adenosylhomocysteine
GSSG: Oxidized glutathione
PC: Phosphatidylcholine
ACh: Acetylcholine
IOM: Institute of Medicine
NDSR: Nutrition Data System for Research
ATN/IDDRC: Autism Treatment Network/Intellectual and Developmental Disabilities Research Center.
